# Sydenham Society of London

**Published:** 1850-12

**Authors:** 


### SYDENHAM SOCIETY OF LONDON.

We are informed that this valuable Society is about to place soon, in the hands of its members, another volume of the excellent work of Rokitansky on Pathological Anatomy.

